# CCR4‐IL2 bispecific immunotoxin is more effective than brentuximab for targeted therapy of cutaneous T‐cell lymphoma in a mouse CTCL model

**DOI:** 10.1002/2211-5463.13625

**Published:** 2023-05-15

**Authors:** Zhaohui Wang, Jihong Ma, Huiping Zhang, Rashmi Ramakrishna, Danielle Mintzlaff, David W. Mathes, Elizabeth A. Pomfret, M. Scott Lucia, Dexiang Gao, Bradley M. Haverkos, Zhirui Wang

**Affiliations:** ^1^ Division of Plastic and Reconstructive Surgery, Department of Surgery, School of Medicine University of Colorado Anschutz Medical Campus Aurora CO USA; ^2^ Division of Transplant Surgery, Department of Surgery, School of Medicine University of Colorado Anschutz Medical Campus Aurora CO USA; ^3^ Department of Pathology, School of Medicine University of Colorado Anschutz Medical Campus Aurora CO USA; ^4^ Department of Pediatrics, University of Colorado Cancer Center Biostatistics and Bioinformatics Shared Resource University of Colorado Anschutz Medical Campus Aurora CO USA; ^5^ University of Colorado Hospital, School of Medicine University of Colorado Anschutz Medical Campus Aurora CO USA

**Keywords:** adcetris, brentuximab vedotin, CCR4‐IL2 bispecific immunotoxin, cutaneous T‐cell lymphoma, diphtheria toxin, immunotoxin

## Abstract

Cutaneous T‐cell lymphoma (CTCL) encompasses two main subtypes: mycosis fungoides and Sezary syndrome. Global response rates for the systemic treatment of mycosis fungoides and Sezary syndrome are approximately 30%, and none of these treatments are thought to be curative. C–C chemokine receptor type 4 (CCR4) and CD25 are encouraging targets for the treatment of CTCL and are individually targeted by mogamulizumab and denileukin diftitox, respectively. We developed a novel CCR4‐IL2 bispecific immunotoxin (CCR4‐IL2 IT) targeting both CCR4 and CD25. CCR4‐IL2 IT demonstrated superior efficacy against CCR4^+^CD25^+^CD30^+^ CTCL in an immunodeficient *NSG* mouse tumor model. Investigative New Drug‐enabling studies of CCR4–IL2 IT are ongoing, including Good Manufacturing Practice production and toxicology studies. In this study, we compared the *in vivo* efficacy of CCR4‐IL2 IT versus the US Food and Drug Administration–approved drug, brentuximab, using an immunodeficient mouse CTCL model. We demonstrated that CCR4–IL2 IT was significantly more effective in prolonging survival than brentuximab, and combination treatment of CCR4–IL2 IT and brentuximab was more effective than brentuximab or CCR4–IL2 IT alone in an immunodeficient NSG mouse CTCL model. Thus, CCR4–IL2 IT is a promising novel therapeutic drug candidate for CTCL treatment.

## Graphical abstract

We have compared the *in vivo* efficacy of CCR4‐IL2 bispecific immunotoxin (CCR4‐IL2 IT) versus the FDA‐approved brentuximab in an immunodeficient mouse CTCL model. We demonstrated that CCR4–IL2 IT was significantly more effective in prolonging survival than brentuximab, and combination treatment of CCR4–IL2 IT and brentuximab was more effective than brentuximab or CCR4–IL2 IT alone.

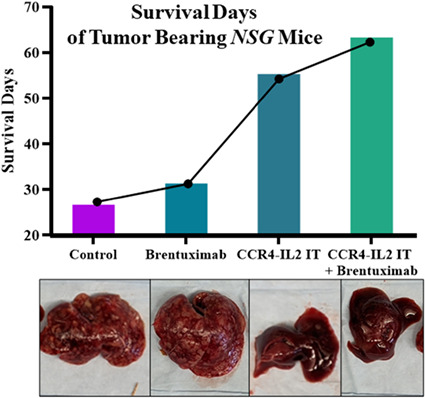

**Abbreviations**
7‐AAD: 7‐amino‐actinomycin D
ATP: adenosine triphosphate
CCR4: C–C chemokine receptor type 4
CDX: cell line‐derived xenograft
CTCL: cutaneous T‐cell lymphoma
DT: diphtheria toxin
FDA: Food and Drug Administration
FITC: Fluorescein isothiocyanate
GLP: good laboratory practice
GMP: good manufacturing practice
IC50: half maximal inhibitory concentration
IL2: interleukin 2
IP: intraperitoneal injection
IT: immunotoxin or fusion toxin
IV: intravenous injection
KD: dissociation constant
*M*
: *molar concentration*
mAb: monoclonal antibody
*MF*
: *mycosis fungoides*
MFI: mean fluorescence intensity
MMAE: monomethyl auristatin E
nM: nanomolar concentration
*NSG*
: *NOD/SCID IL‐2 receptor γ−/−*
PDX: Patient‐derived xenograft
PE: phycoerythrin
scFv: single‐chain variable fragment
SD: standard deviation
SS: Sezary syndrome
Tregs: regulatory T cells

Cutaneous T‐cell lymphoma (CTCL) is a heterogeneous subset of extranodal non‐Hodgkin's lymphoma characterized by skin lesions resulting from the infiltration of malignant T lymphocytes. The two main forms of CTCL are mycosis fungoides (MF) and Sezary syndrome (SS) [1, 2]. The incidence of CTCL is ~ 3000 new cases per year with an estimated prevalence of 24 000 in the United States in 2016. Treatment of early‐stage CTCL (IA‐IIA) primarily involves the use of skin‐directed therapies involving topical corticosteroids, phototherapy, topical chemotherapy, topical bexarotene, and radiotherapy, including localized radiation and total skin electron beam therapy [3, 4, 5]. Refractory early‐ and advanced‐stage CTCL (IIB‐IV) requires systemic treatment, such as bexarotene (a retinoid), vorinostat (an inhibitor of histone deacetylases), denileukin diftitox (truncated diphtheria toxin‐based IL2 fusion toxin), romidepsin (a natural product from a bacterium that blocks histone deacetylases), brentuximab (an anti‐CD30 antibody–drug conjugate), and a recently approved mogamulizumab [a humanized, afucosylated monoclonal antibody targeting the C–C chemokine receptor type 4 (CCR4) receptor]. Allogeneic bone marrow transplantation is a potential curative treatment option for eligible patients. This is particularly efficacious in Sezary syndrome and provides a strong evidence for the benefit of immunotherapy for cancer treatment. CD25, CCR4, and CD30 are highly expressed on the CTCL surface. These surface markers are thus recognized as ideal targets for CTCL therapy. The objective response rate remains approximately 30% with current treatment strategies for relapsed/refractory CTCL [6, 7]. Therefore, a significant unmet medical need remains for the development of novel‐targeted therapeutic drug candidates for the treatment of relapsed/refractory CTCL.

Brentuximab vedotin (Adcetris^®^, Seagen Inc., Bothell, WA, USA) is an antibody–drug conjugate consisting of a mouse human chimeric anti‐CD30 monoclonal antibody (mAb) conjugated to the antimitotic agent, monomethyl auristatin E (MMAE), with a protease‐sensitive peptide linker. Brentuximab is selectively delivered to CD30^+^ cells where the cytotoxic compound is released intracellularly and causes cell‐cycle arrest by interfering with microtubule formation [8, 9]. Between 12% and 23% of MF/SS cases expressed CD30. Brentuximab was approved by the US Food and Drug Administration (FDA) for CTCL treatment in 2017, and it is now a leading drug in the CTCL clinical market.

Mogamulizumab (Poteligeo^®^, Kyowa Kirin, Tokyo, Japan) is a humanized, afucosylated mAb that targets CCR4. In 2014, mogamulizumab was approved for the treatment of CCR4^+^ relapsed/refractory CTCL in Japan and in 2018 in the United States. The overall response rate was 36.8% [10]. However, the MAVORIC study demonstrated that the response rates of Mogamulizumab were low in solid organs such as lymph nodes (17%) and skin (48%), not as in the peripheral blood (68%). The response rates were also different in MF (21%) and SS (37%) [11]. The therapeutic effect of mAbs, including mogamulizumab, relies on accessory cells from the innate immune system to initiate antibody‐dependent cellular cytotoxicity, complement‐dependent cytotoxicity, or antibody‐dependent cellular phagocytosis. Often heavily pretreated patients with cancer have poor accessory cell function and can therefore easily develop resistance to therapeutic antibodies. Therefore, novel therapeutics are required to be developed for patients with mogamulizumab‐resistant CCR4^+^ CTCL.

Denileukin diftitox (Ontak^®^) is a truncated diphtheria toxin based recombinant human IL2 fusion toxin that was approved in 1999 by the FDA for the treatment of patients with recurrent CD25^+^ CTCL. The overall response rates to denileukin diftitox range from 30% to 50% [1, 2]. Unfortunately, denileukin diftitox was discontinued clinically in 2014 due to the low quality of its purification related to the *Escherichia coli* expression system used. E7777 is a new version of denileukin difftitox with improved purity and an increased percentage of active monomer. Phase II clinical trial [12] demonstrated that the objective response rate was 36% and the median progression‐free survival was 3.1 months.

Recently, we have developed a CCR4‐IL2 bispecific immunotoxin (CCR4‐IL2 IT) using a unique diphtheria toxin‐resistant *Pichia pastoris* yeast expression system. Our yeast expression system overcomes expression and purification problems encountered with *E. coli*‐based expression systems to deliver a high level of production and excellent purification of CCR4‐IL2 IT. CCR4‐IL2 IT demonstrated superior efficacy against CCR4^+^CD25^+^CD30^+^ Hut102/6TG CTCL in an immunodeficient *NSG* mouse tumor model [13]. Investigative New Drug‐enabling studies, including Good Manufacturing Practice production and toxicology studies, are ongoing. In this study, we compared the *in vivo* efficacy of CCR4‐IL2 IT versus the FDA‐approved leading drug, brentuximab, for targeted therapy of CTCL using an immunodeficient *NSG* mouse tumor model.

## Materials and methods

### Immunotoxins, brentuximab, antibodies, and tumor cell line

CCR4–IL2 bispecific immunotoxin [13], single‐chain fold‐back diabody antihuman CCR4 immunotoxin (CCR4 IT) [14], Ontak^®^‐like human IL2 fusion toxin (IL2 IT) [15, 16] and C21 immunotoxin (C21 IT) were all made in our laboratory using a unique diphtheria toxin‐resistant *Pichia pastoris* yeast expression system [17]. Pharmaceutical grade brentuximab vedotin (Adcetris^®^) was used in this study. A human CD25^+^CCR4^+^CD30^+^ CTCL Hut 102/6TG cell line (RRID: CVCL_3526) [18] was generously provided by Dr. Robert Harrison from Anjin Group, Inc., Boston, MA, USA. Phycoerythrin (PE)‐mouse antihuman CD30 antibody (#333906), fluorescein isothiocyanate (FITC)‐mouse antihuman CD25 antibody (#302603), and 7‐amino‐actinomycin D (7‐AAD) were purchased from BioLegend (San Diego, CA, USA). FITC‐mouse antihuman CCR4 antibody (#FAB1567F) was purchased from R&D Systems (Minneapolis, MN, USA).

### *In vitro* binding affinity analysis

Human CD25^+^CCR4^+^CD30^+^ Hut102/6TG cells were stained with Alexa Fluor 488‐labeled CCR4‐IL2 IT or brentuximab using a wide range of concentrations (0.1–200 nm). Fluorescein‐mouse antihuman/rat CCR4 mAb (26 nm), FITC‐mouse antihuman CD25 mAb (50 nm), and PE‐mouse antihuman CD30 mAb (25 nm) were included as positive controls. Unstained cells were used as a negative control. Flow cytometry was carried out using a CytoFLEX Flow cytometer (Beckman Coulter, Brea, CA, USA), and data were analyzed using the flowjo software (Flowjo, LLC, Ashland, OR, USA).

### *K*_D_ determination

A binding affinity comparison of brentuximab versus CCR4‐IL2 IT for human CD25^+^CCR4^+^CD30^+^ Hut102/6TG cells was performed using a wide range of concentrations of the Alexa Fluor 488‐labeled CCR4‐IL2 IT and brentuximab. A dissociation constant (*K*_D_) determination was performed using nonlinear regression analysis of the flow cytometry data with a saturation binding equation (graphpad prism 9.4.1, GraphPad Software, San Diego, CA, USA). The median fluorescence intensity (MFI) was plotted versus the concentrations of the Alexa Fluor 488‐labeled brentuximab or CCR4‐IL2 IT. Nonlinear regression analysis was based on the eq. *Y* = *B*_max_ × *X*/(*K*_D_ + *X*), where *Y* = MFI at a given concentration of Alexa Fluor 488‐labeled brentuximab or CCR4‐IL2 IT after subtracting the background, *X* = the concentration of the Alexa Fluor 488‐labeled brentuximab or CCR4‐IL2 IT, and *B*_max_ = the maximum specific binding in the same units as *Y*.

### *In vitro* efficacy

A comparison of the *in vitro* efficacy of brentuximab versus CCR4‐IL2 IT in the inhibition of the viability of human CD25^+^CCR4^+^CD30^+^ CTCL Hut102/6TG cells was performed using a CellTiter‐Glo^®^ Luminescent Cell Viability Assay (Promega, Madison, WI, USA) as described previously [19]. This assay measures the luminescence produced by ATP production from metabolically active cells. Increasing concentrations of brentuximab or CCR4‐IL2 IT induce cell death and a corresponding reduction in ATP‐related fluorescence. Luminescence signals were measured using a BioTek Synergy LX Multi‐Mode Reader. C21 IT, Ontak^®^‐like IL2‐IT, and CCR4 IT were also included as negative or monospecific immunotoxin controls, respectively.

### *In vivo* efficacy

Immunodeficient *NSG* mice were purchased from Jackson Laboratories (Bar Harbor, ME, USA). The *in vivo* experiments were approved by the University of Colorado Anschutz Medical Campus Animal Care and Use Committee. As shown in Table 1, 6–8‐week‐old *NSG* mice were divided into the following seven groups: (a) CCR4‐IL2 IT (*n* = 7); (b) CCR4 IT as a monospecific immunotoxin control targeting CCR4 receptor (*n* = 7); (c) Ontak^®^‐like IL2 IT as another monospecific immunotoxin control targeting CD25 (*n* = 7); (d) brentuximab full‐dose (*n* = 7) based on Bhatt et al. [20]; (e) Combination treatment with CCR4‐IL2 IT and a brentuximab full‐dose (*n* = 7); (f) C21 IT as a negative control (a nonrelated truncated diphtheria toxin‐based immunotoxin) (*n* = 7); and (g) brentuximab matching‐dose (exactly the same molar dose as the imuunotoxins) group (*n* = 7). All animals were intravenously injected on Day 0 with 1.0 × 10^7^ human CD25^+^CCR4^+^CD30^+^ Hut102/6TG tumor cells via tail veins. The immunotoxin (CCR4‐IL2 IT, or CCR4 IT, or IL2 IT or C21 IT) or brentuximab matching‐doses were intraperitoneally (IP) injected starting on Day 4 at 8.43 × 10^−10^ moles·kg^−1^, once daily for 10 consecutive days (10 doses in total). This dose was based on our previous publication [13]. A brentuximab full‐dose was IP injected starting on Day 4 at 3 mg·kg^−1^, once every other day for 10 consecutive days (5 doses in total). For combination treatment, CCR4‐IL2 IT was IP injected at 8.43 × 10^−10^ moles·kg^−1^, once daily for 10 consecutive days (10 doses in total) and a brentuximab full‐dose was IP injected at 3 mg·kg^−1^, once every other day for 10 days (5 doses in total). Tumor‐bearing mice were observed daily for signs and symptoms of illness and scored at least twice weekly based on parameters as previously reported by our laboratory [14, 21]. The animals were humanely euthanized when the body condition score exceeded the limit [21], or the animal lost more than 15% of its pre‐injection body weight.

**Table 1:** Dosing schedule of *in vivo* efficacy of CCR4‐IL2 IT versus brentuximab.

Group	Mice number	Drug name	Dose (μg·kg^−1^)	Molar dose (molar·kg^−1^)	Drug IP injection day (post‐tumor cell injection)										
					0	4	5	6	7	8	9	10	11	12	13
CCR4‐IL2 IT	7	CCR4‐IL2 IT	73	8.43E‐10	CTCL	X	X	X	X	X	X	X	X	X	X
CCR4 IT	7	CCR4 IT	81.2	8.43E‐10	CTCL	X	X	X	X	X	X	X	X	X	X
IL2 IT	7	IL2 IT	50	8.43E‐10	CTCL	X	X	X	X	X	X	X	X	X	X
Brentuximab‐full dose	7	Brentuxi‐mab	3000	1.96E‐08	CTCL	X		X		X		X		X	
Combined treatment	7	CCR4‐IL2 IT	73	8.43E‐10	CTCL	X	X	X	X	X	X	X	X	X	X
		Brentuxi‐mab	3000	1.96E‐08		X		X		X		X		X	
Negative control	7	C21 IT	59	8.43E‐10	CTCL	X	X	X	X	X	X	X	X	X	X
Brentuximab matching	7	Brentuxi‐mab	129	8.43E‐10	CTCL	X	X	X	X	X	X	X	X	X	X

### Pathology analysis

Liver necropsy specimens were harvested surgically on Day 28 after animal euthanasia. Tissues were fixed in 10% formalin, embedded in paraffin, and subsequently sectioned. Tissues were stained with hematoxylin and eosin for routine light microscopy. Slides were digitalized by an aperio scanscope (Leica, Wetzlar, Germany), and images were analyzed at 1.4× and 30× with the aperio imagescope software (Leica).

### Statistical analysis

The half maximal inhibitory concentration (IC_50_) was determined using nonlinear regression analysis (graphpad prism 9.4.0). Kaplan–Meier survival curves were displayed for the survival functions of the treatment groups for the *in vivo* experiments. The primary comparison for the survival experiments was between CCR4‐IL2 IT and brentuximab full‐dose. The secondary comparisons included the combination versus Brentuximab and the combination versus CCR4‐IL2 IT. Treatment effects on overall survival were estimated using a frailty Cox proportional hazard regression model, where the measures from the two sets of experiments in this study were combined, and a random effect was included in the model to account for the potential correlations among measures within each set of the experiments. Sample size/power consideration: The study is powered on the primary comparison between CCR4‐IL2 IT and brentuximab full‐dose. Seven mice per group provide 80% power to detect a hazard ratio of 0.26 in favor of CCR4‐IL2 IT using a one‐sided test at a 0.05 significance level. All the mice will be followed till euthanasia. The *P*‐values for survival curve comparisons were calculated using a Mantel–Cox log‐rank test (graphpad prism 9.4.1). *P* < 0.05 was considered statistically significant. sas software 9.4 (SAS Institute Inc., Cary, NC, USA) was used to carry out the frailty Cox proportional hazard regression analysis.

## Results

### *In vitro* binding affinity and efficacy of CCR4‐IL2 IT versus brentuximab

**Fig. 1:**
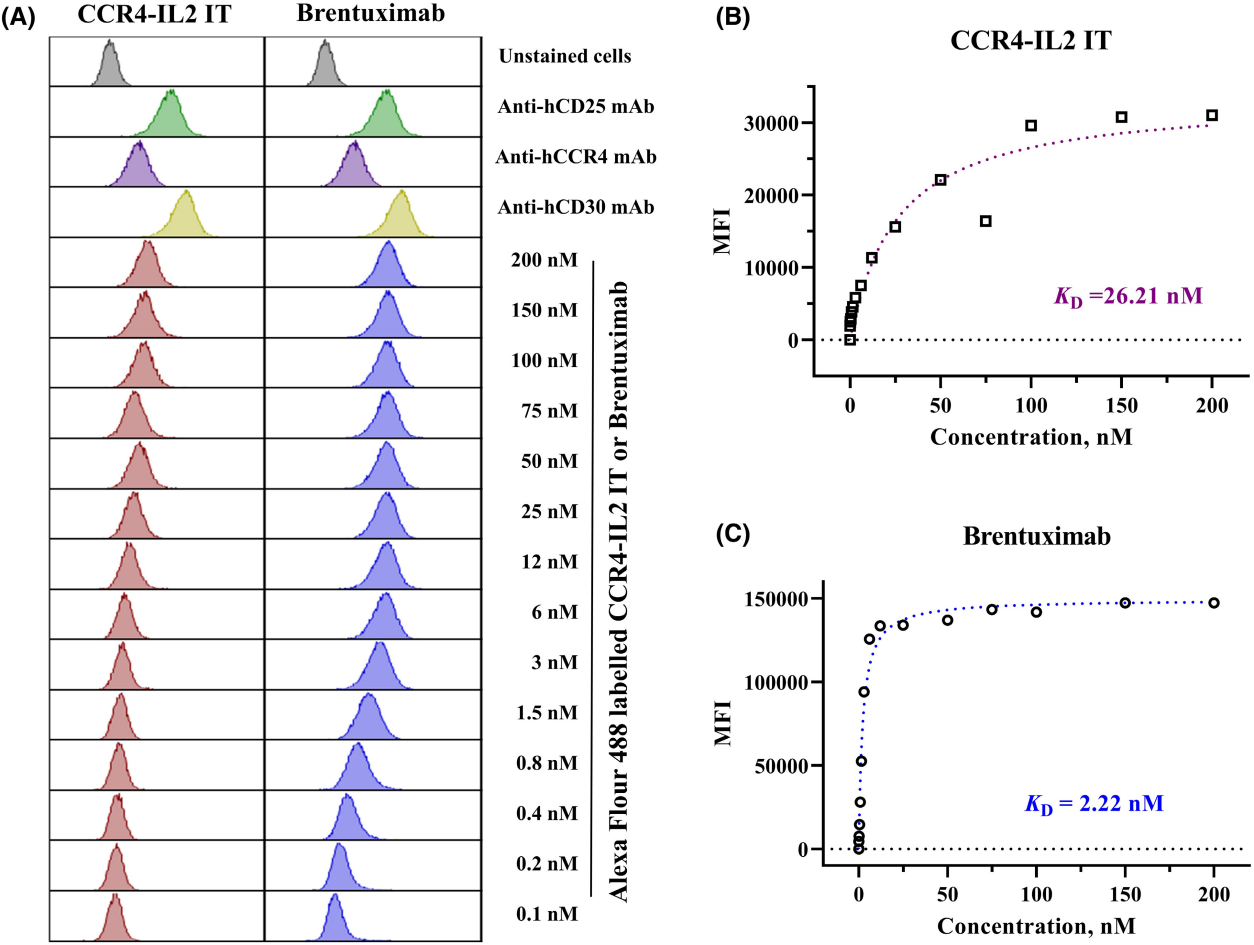
*In vitro* binding affinity of CCR4‐IL2 IT versus brentuximab. (A) Flow cytometry binding affinity analysis of Alexa Fluor 488–labeled CCR4‐IL2 IT or brentuximab and human CD25^+^CCR4^+^CD30^+^ Hut102/6TG cells. Fluorescein‐mouse antihuman/rat CCR4 mAb, FITC‐mouse antihuman CD25 mAb, and PE‐mouse antihuman CD30 mAb were included as positive controls. The data are representative of three individual experiments. (B–C) *K*_D_ comparison of CCR4‐IL2 IT and brentuximab. *K*_D_ was determined using nonlinear regression analysis of the flow cytometry data with a saturation binding equation (graphpad prism 9.4.1). The MFI was plotted over a wide range of concentrations of the Alexa Fluor 488–labeled CCR4‐IL2 IT or brentuximab. Nonlinear regression analysis was based on the eq. *Y* = *B*_max_ × *X*/(*K*_D_ + *X*), where *Y* = MFI at a given concentration of Alexa Fluor 488–labeled brentuximab or CCR4‐IL2 IT after subtracting the background, *X* = the concentration of the Alexa Fluor 488–labeled brentuximab or CCR4‐IL2 IT, and *B*_max_ = the maximum specific binding in the same units as *Y*. CCR4‐IL2 IT, C–C chemokine receptor type 4 interleukin 2 bispecific immunotoxin; FITC, fluorescein isothiocyanate; *K*_D_, dissociation constant; mAb, monoclonal antibody; MFI, median fluorescence intensity; PE, phycoerythrin.

**Fig. 2:**
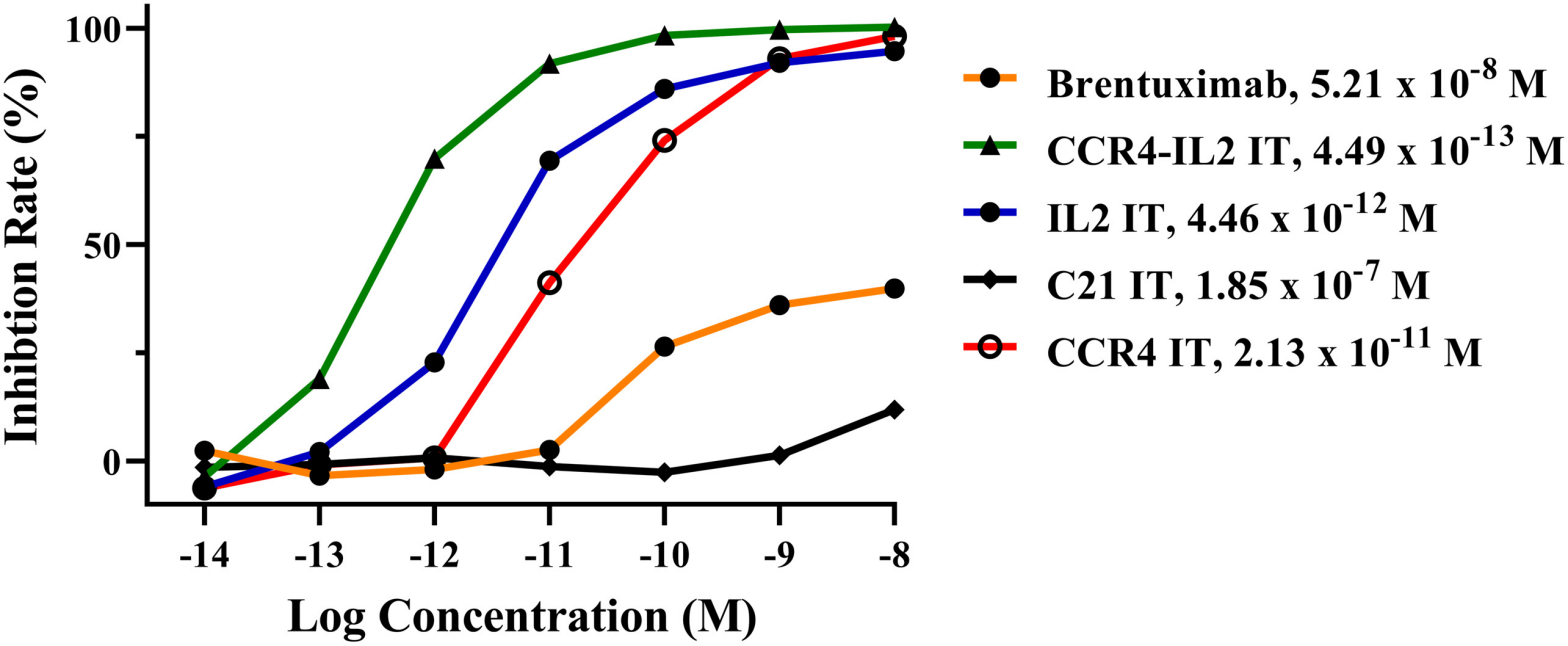
*In vitro* efficacy of CCR4‐IL2 IT versus brentuximab in the human CD25^+^CCR4^+^CD30^+^ T‐cell lymphoma cell line, Hut102/6TG. A CellTiter‐Glo® Luminescent Cell Viability Assay (Promega, cat# G7571) was used. Brentuximab (orange line), CCR4‐IL2 IT (green line), Ontak®‐like IL2‐IT (blue line), C21 IT as negative immunotoxin control (black line), CCR4 IT (red line). Y‐axis: inhibition rate of cell viability by determining the number of viable cells based on quantification of the ATP present. *X*‐axis: plated immunotoxin concentration. Cycloheximide (1.25 mg·mL^−1^) was used as a positive control. The negative control consisted of cells without immunotoxin. Data are representative of three assays. C21 IT, C21 immunotoxin; CCR4 IT, C–C chemokine receptor type 4 immunotoxin; CCR4‐IL2 IT, CCR4‐interleukin 2 bispecific immunotoxin; IL2 IT, IL2 fusion toxin.

As shown in Fig. 1, we compared the *in vitro* binding affinity of CCR4‐IL2 IT versus brentuximab to CD25^+^CCR4^+^CD30^+^ Hut102/6TG cells using flow cytometry analysis. *In vitro* binding affinity data demonstrated that brentuximab (*K*_D_ = 2.22 nm) bound significantly more strongly than CCR4‐IL2 IT (*K*_D_ = 22.61 nm). A comparison of the *in vitro* efficacy of CCR4‐IL2 IT versus brentuximab to human CD25^+^CCR4^+^CD30^+^ Hut102/6TG cells was performed using a luminescent‐based cell viability assay. We demonstrated that CCR4‐IL2 IT (IC_50_ = 4.49 × 10^−13^ m) was significantly more potent than brentuximab (IC_50_ = 5.21 × 10^−8^ m). Ontak^®^‐like IL2 IT (IC_50_ = 4.46 × 10^−12^ m) and CCR4 IT (IC_50_ = 2.13 × 10^−11^ m) were also included as monospecific immunotoxin controls. C21 IT (IC_50_ = 1.85 × 10^−7^ m) was included as a negative immunotoxin control (Fig. 2).

### *In vivo* efficacy of CCR4‐IL2 IT versus brentuximab

**Fig. 3:**
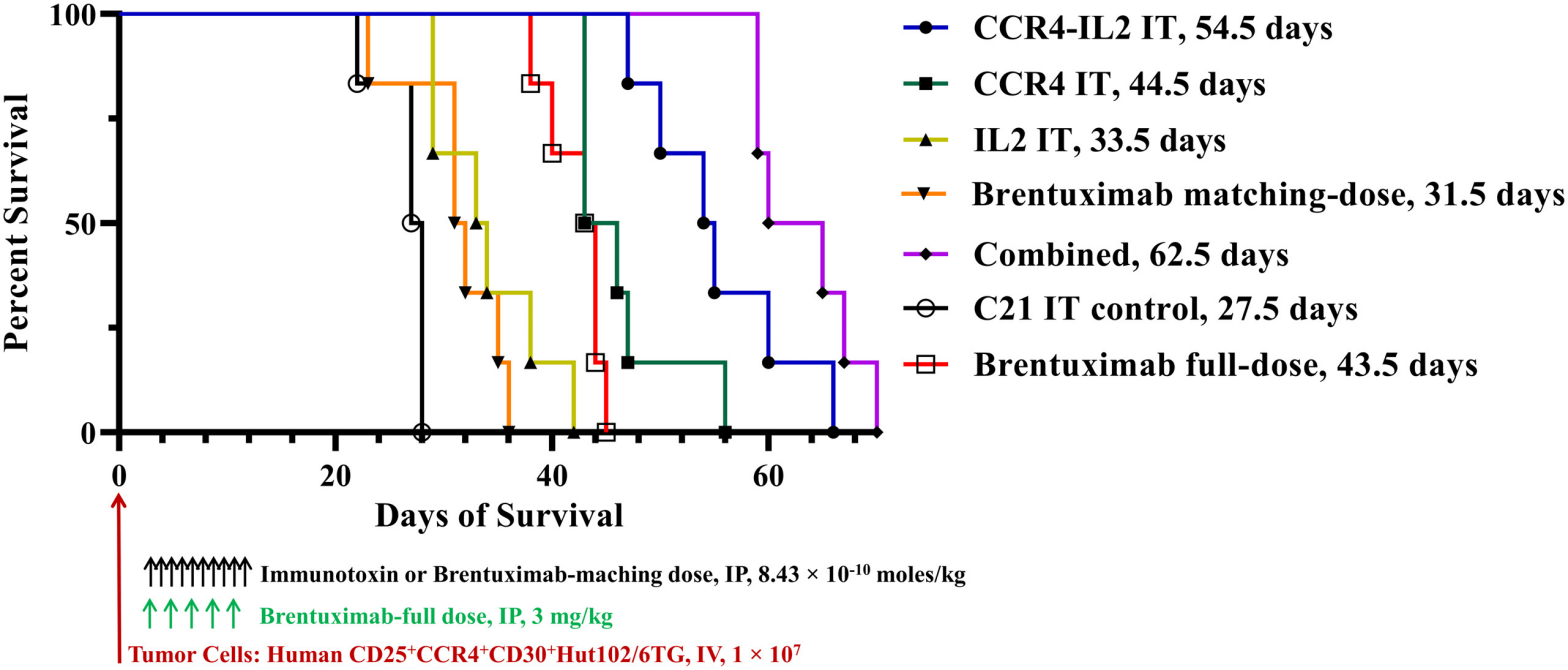
*In vivo* efficacy comparison of CCR4‐IL2 IT versus brentuximab in an immunodeficient mouse tumor model. Immunodeficient *NSG* mice were IV injected with 1.0 × 10^7^ CD25^+^CCR4^+^CD30^+^ Hut102/6TG cells on Day 0. Immunotoxin (CCR4‐IL2 IT, CCR4 IT, IL2 IT or C21 IT) or brentuximab‐matching doses were IP injected starting on day 4 at 8.43 × 10^−10^ moles·kg^−1^, once daily for 10 consecutive days (10 doses in total). A brentuximab full dose was IP injected starting on day 4 at 3 mg·kg^−1^, once every other day for 10 consecutive days (5 doses in total). For combination treatment, CCR4‐IL2 IT was IP injected at 8.43 × 10^−10^ moles·kg^−1^, once daily for 10 consecutive days (10 doses in total) and a brentuximab full dose was IP injected at 3 mg·kg^−1^, once every other day for 10 days (5 doses in total). CCR4‐IL2 IT group (*n* = 7, blue curve) with a median survival time of 54.5 days. CCR4 IT group (*n* = 7, green curve) with a median survival time of 44.5 days. IL2 IT group (*n* = 7, light yellow curve) with a median survival time of 33.5 days. Brentuximab matching‐dose group (*n* = 7, orange curve) with a median survival time of 31.5 days. Combination treatment of CR4‐IL2 IT and brentuximab full‐dose groups (*n* = 7, purple curve) with a median survival time of 62.5 days. C21 IT group (a non‐related DT390‐based immunotoxin as a negative control) (*n* = 7, black curve) with a median survival time of 27.5 days. Brentuximab full‐dose group (*n* = 7, red curve) with a median survival time of 43.5 days. The schedules for the IP injection of immunotoxin or brentuximab, and IV injection of tumor cells, are pictured in the schematic below the survival curve. The vertical arrows indicate the days on which tumor cells (red arrows), immunotoxin or brentuximab matching‐dose (black arrows), or brentuximab‐full dose (green arrows) were injected. C21 IT, C21 immunotoxin; CCR4 IT, C–C chemokine receptor type 4 immunotoxin; CCR4‐IL2 IT, CCR4‐interleukin 2 bispecific immunotoxin; IP, intraperitoneally; IL2 IT, IL2 fusion toxin; IV, intravenously.

We compared CCR4‐IL2 IT versus brentuximab using an immunodeficient *NSG* mouse tumor model. As shown in Table 1, 10 million human CD25^+^CCR4^+^CD30^+^ Hut102/6TG cells were IV injected into immunodeficient *NSG* mice via tail veins on Day 0. Immunotoxin (CCR4‐IL2 IT, or CCR4 IT, or IL2 IT or C21 IT) or brentuximab matching‐dose treatment was started on Day 4 at 8.43 × 10^−10^ moles·kg^−1^, once daily for 10 consecutive days. For a brentuximab full‐dose, this was given at 3 mg·kg^−1^, once every other day for 10 days (5 doses in total). For combination treatment, CCR4‐IL2 IT was IP injected at 8.43 × 10^−10^ moles·kg^−1^, once daily for 10 consecutive days (10 doses in total) and a brentuximab full‐dose was IP injected at 3 mg·kg^−1^, once every other day for 10 consecutive days (5 doses in total). The primary efficacy endpoint of this study is survival. As shown in Fig. 3, Kaplan–Meier survival curves demonstrated that the survival of tumor‐bearing animals in the CCR4‐IL2 IT group was significantly longer than for animals of both brentuximab full‐dose and brentuximab matching‐dose groups. Animals of the combination treatment group survived longer than those of either the CCR4‐IL2 IT or brentuximab alone groups. In addition, we also demonstrated that the survival of animals of the brentuximab full‐dose group was comparable with that of animals in the CCR4 IT control group indicating that the *in vivo* efficacy of a full dose of brentuximab is comparable with CCR4 IT. The results from the frailty Cox proportional regression model were consistent with the above results (Table 2). Compared to mice treated with brentuximab full‐dose, the hazards of death for mice treated with CCR4‐IL2 IT and the combination are only 6.5% (95% CI, 2.2%, 19.5%) and 3% (95% CI, 0.9%–9.6%), respectively, of the hazard of those treated with brentuximab full‐dose. In addition, the hazard of death for mice treated with the combination is 45.5% of the hazard for mice treated with CCR4‐IL2 IT with a marginal significance (*P* = 0.058). These results indicated both CCR4 IT and the combination prolong mice survival significantly compared to brentuximab, and the combination is the most effective.

**Table 2:** Frailty Cox regression model results. The model was carried out on the combined datasets of the *in vivo* experiments, and a random effect was included in the model to account for the potential correlations among measures within each set of experiments.

Comparison	HR	Standard error	95% CI		*P* value
			Lower limit	Upper limit	
Primary					
CCR4‐IL2 IT versus Brentuximab full	0.065	0.037	0.022	0.20	< 0.0001
Secondary					
CCR4‐IL2 IT versus C21 IT	0.006	0.004	0.002	0.021	< 0.0001
Combination versus Brentuximab full	0.030	0.018	0.009	0.096	< 0.0001
Combination versus CCR4‐IL2 IT	0.455	0.189	0.201	1.029	0.0585

**Fig. 4:**
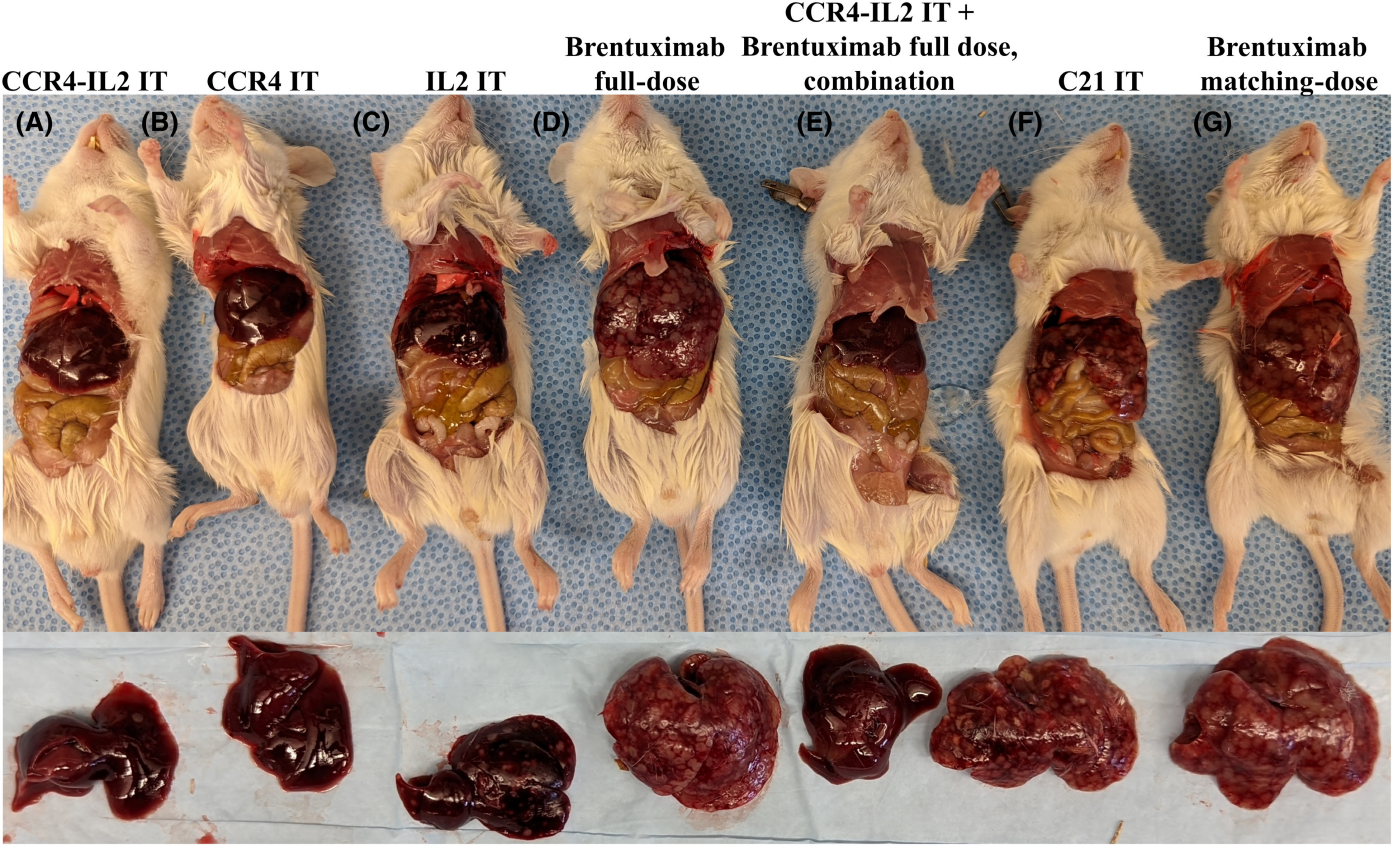
Liver necropsy gross examination of representative tumor‐bearing mice on day 28. (A) CCR4‐IL2 IT group. (B) CCR4 IT group. (C) Ontak®‐like IL2 IT group. (D) Brentuximab full‐dose group. (E) Combination of CR4‐IL2 IT and brentuximab full‐dose groups. (F) C21 IT group. (G) Brentuximab matching‐dose group. C21 IT, C21 immunotoxin; CCR4 IT, C–C chemokine receptor type 4 immunotoxin; CCR4‐IL2 IT, CCR4‐interleukin 2 bispecific immunotoxin; IL2 IT, IL2 fusion toxin.

On Day 28, we simultaneously euthanized a representative tumor‐bearing animal from each treatment group for gross examination and pathology analysis. Gross examination (Fig. 4) revealed that liver sizes were normal for CCR4‐IL2 IT‐, CCR4 IT‐, and combination‐treated animals. We did not observe white tumor nodules on the liver surfaces of the combination‐treated animals. We observed a few white tumor nodules on the liver surfaces of CCR4‐IL2 IT‐ and CCR4 IT‐treated animals. In contrast, we observed enlarged livers and extensive white tumor nodules on the liver surfaces of C21 IT, brentuximab full‐dose, and brentuximab matching‐dose animals. We also observed some white tumor nodules on the liver surfaces of Ontak^®^‐like IL2 IT‐treated animals. As shown in Fig. 4, we did not observe a difference in liver size and white tumor nodule amount on the liver surface between brentuximab full‐dose– and brentuximab matching‐dose–treated animals. We hypothesized that Day 28 was too late to show a difference between brentuximab full‐dose– and brentuximab matching‐dose–treated animals. Therefore, we conducted a second gross liver examination on Day 22 during the repeated *in vivo* efficacy comparison study. A difference of the white tumor nodule amount was observed between brentuximab full‐dose and brentuximab matching‐dose animals. As expected, a brentuximab full‐dose was more effective in suppressing the tumor metastasis to the liver than a brentuximab matching‐dose and a brentuximab matching‐dose was more effective than a negative control C21 IT (Fig. S1).

**Fig. 5:**
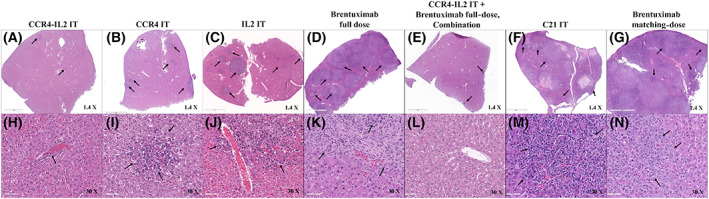
Liver pathology analysis of representative tumor‐bearing mice on Day 28. (A, H) Liver from a mouse injected with both Hut102/6TG tumor cells and CCR4‐IL2 IT shows a few small, scattered tumor nests in the examined section. (B, I) Liver from a mouse injected with both Hut102/6TG tumor cells and CCR4 IT shows some small tumor cell nests in the examined section. (C, J) Liver from a mouse injected with both Hut102/6TG tumor cells and IL2 IT shows larger tumor foci in the examined section. (D, K) Liver from a mouse injected with both Hut102/6TG tumor cells and a brentuximab full dose shows numerous large tumor foci in the examined section. (E, L) Liver from a mouse injected with both Hut102/6TG tumor cells and the combination of CCR4‐IL2 IT and a brentuximab full dose shows normal hepatic parenchyma without tumor in the examined section. (F, M) Liver from a mouse injected with both Hut102/6TG tumor cells and C21 IT (negative control) shows extensive tumor infiltration with replacement of liver parenchyma by tumor cells. (G, N) Liver from a mouse injected with both Hut102/6TG tumor cells and a brentuximab matching dose also shows extensive tumor cell areas in the examined section. Scale bars of panel A–G: 2 mm and panel H–N: 70 μm. C21 IT, C21 immunotoxin; CCR4 IT, C–C chemokine receptor type 4 immunotoxin; CCR4‐IL2 IT, CCR4‐interleukin 2 bispecific immunotoxin; IL2 IT, IL2 fusion toxin.

Pathological analysis demonstrated a complete lack of tumors in the examined liver sections of combination‐treated animals (Fig. 5E,L). Only a few small tumor cell nests were observed in the livers of CCR4‐IL2 IT‐treated animals (Fig. 5A,H). In contrast, the tumor cell burden was mildly increased in CCR4 IT‐treated animals (Fig. 5B,I), and moderately increased in Ontak^®^‐like IL2 IT‐treated animals (Fig. 5C,J). The tumor burden was much higher in brentuximab full‐dose– (Fig. 5D,K), brentuximab matching‐dose (Fig. 5G,N), and C21 IT (Fig. 5F,M)–treated animals, with extensive replacement of normal liver tissues by tumor. Gross examination and pathology data supported Kaplan–Meier survival curves. Taken together, all *in vivo* data above demonstrated that CCR4‐IL2 IT is more effective in prolonging survival of the tumor‐bearing animals and suppressing tumor metastasis to the livers than brentuximab. Combination of CCR4‐IL2 IT and brentuximab are more effective than either CCR4‐IL2 IT or brentuximab alone.

Brentuximab is an antibody–drug conjugate that is required to be stored at 4 °C. To rule out any concerns about drug stability, we repeated the *in vivo* efficacy comparison study using fresh brentuximab and obtained similar results (Fig. S2).

## Discussion

It is novel that we have demonstrated that CCR4‐IL2 IT was remarkably more effective in a mouse CTCL model than brentuximab, an FDA‐approved leading drug in CTCL clinical market. We have also demonstrated that the combination treatment of CCR4‐IL2 IT and brentuximab were more effective than either CCR4‐IL2 IT or brentuximab alone. Therefore, we believe that CCR4‐IL2 IT will have strong competitive advantage in the future CTCL therapy market.

Tregs are one of the main contributors to the generation of an immunosuppressive microenvironment surrounding tumors. Scientists are exploring different novel strategies to deplete tumor‐infiltrating effector Tregs to boost the host's antitumor immune response. CCR4 and CD25 are highly expressed on tumor‐infiltrating effector Tregs [22, 23, 24, 25, 26]. CCR4‐IL2 IT shows strong potential to deplete tumor‐infiltrating effector Tregs and is currently under investigation for a broad‐spectrum cancer treatment via a Treg depletion mechanism. For CTCL treatment, CCR4‐IL2 IT is thought of as hitting “two birds with one stone,” as it will not only deplete CCR4^+^ and/or CD25^+^ CTCL, but also deplete CCR4^+^ and/or CD25^+^ tumor‐infiltrating effector Tregs to boost the overall effect of treatment. CCR4‐IL2 IT can also be a powerful adjuvant for broad‐spectrum cancer treatment by depleting CCR4^+^ and/or CD25^+^ tumor‐infiltrating effector Tregs.

This study indicates that CCR4‐IL2 IT may be used for the treatment of patients with CD30‐negative and brentuximab‐relapsed CTCL as well as combination treatment for patients with CD30^+^CCR4^+^ and/or CD25^+^ CTCL. In this study, we also demonstrated that brentuximab is comparable with CCR4 IT and that CCR4 IT is more effective in prolonging survival of the tumor‐bearing animals than Ontak^®^‐like IL2 IT. We demonstrated that combination treatment of CCR4‐IL2 IT and brentuximab was significantly more effective than brentuximab alone. However, combination treatment was not significantly more effective compared to CCR4‐IL2 IT alone (*P* = 0.073) (Table 3). Although not statistically significant, combination treatment was markedly more effective than CCR‐IL2 IT alone (Fig. 3).

**Table 3:** Significance analysis of survival curves.

	C21 IT	Brentuximab‐matching	IL2 IT	Brentuximab‐full	CCR4 IT	CCR4‐IL2 IT	Combination
C21 IT	‐	*	**	**	**	**	**
Brentuxi‐match	*	‐	*P* = 0.33	***	***	***	***
IL2 IT	**	*P* = 0.33	‐	**	***	***	***
Brentuxi‐full	**	***	**	‐	*P* = 0.095	***	***
CCR4 IT	**	***	***	*P* = 0.095	‐	*	***
CCR4‐IL2 IT	**	***	***	***	*	‐	*P* = 0.073
Combination	**	***	***	***	***	*P* = 0.073	‐

* *P* < 0.05

** *P* < 0.01

*** *P* < 0.001.

As shown in Fig. 2, the *in vitro* efficacy of brentuximab is very low (IC_50_ = 5.21 × 10^−8^ m). However, the *in vivo* efficacy of brentuximab is still excellent. We speculate that the long half‐life of brentuximab as an antibody–drug conjugate may, in part, have contributed to its *in vivo* efficacy. In addition, in this study, same molecules of CCR4‐IL2 IT and brentuximab were used to compare their *in vivo* efficacy in the mouse CTCL model. However, treatment with an equimolar dose of CCR4‐IL2 IT and brentuximab could not guarantee the same molecules of the active monomethyl auristatin E (MMAE) of brentuximab and diphtheria toxin A chain of CCR4‐IL2 IT.

One limitation of this study is that we could not compare the CCR4‐IL2 IT with mogmulizumab in the used immunodeficient mouse CTCL model. We plan to compare the CCR4‐IL2 IT with mogmulizumab in a humanized mouse CTCL model later. Another limitation of this study is that we only used one CTCL cell line Hut102/6TG to compare the efficacy between CCR4‐IL2 IT and brentuximab. Therefore, we have recently obtained another CTCL cell line HH (ATCC, Manassas, VA, USA) to further compare the efficacy between CCR4‐IL2 IT and brentuximab. The investigation is ongoing. We also plan to further compare the efficacy between CCR4‐IL2 IT and brentuximab in patient‐derived xenograft (PDX)‐based mouse CTCL models later. A major and unresolved issue for various forms of targeted therapy for CTCL is the threshold of positivity for the targeted antigen that is required for the efficacy. For example, the brentuximab studies used a 10% cutoff, and the denileukin diftitox studies used a 20% cutoff. In this cell‐line‐derived xenograft (CDX) based mouse CTCL model, targeted antigen should be expressed on all CTCL Hut102/6TG cells. Therefore, this mouse CTCL model has limitation to recapitulate all features of the CTCL patients including threshold of positivity for the targeted antigen. It is needed to confirm the experimental results in the CTCL patients during the clinical trials.

## Conclusion

We have demonstrated that CCR4‐IL2 IT was more effective in prolonging survival than the FDA‐approved CTCL leading drug, brentuximab, in an immunodeficient *NSG* mouse CTCL model. Combination treatment with CCR4‐IL2 IT and brentuximab was more effective than brentuximab or CCR4‐IL2 IT alone. CCR4‐IL2 IT is a promising novel targeted therapeutic drug candidate for the treatment of relapsed and refractory CTCL.

## Conflict of interest

Zhirui Wang is founder of Rock Immune LLC. All other authors declare no conflict of interest.

### Peer review

The peer review history for this article is available at https://www.webofscience.com/api/gateway/wos/peer‐review/10.1002/2211‐5463.13625.

## Author contributions

Zhaohui W, JM, and HZ primarily performed the experiments, data analysis, and participated in writing the manuscript. RR and DM participated in the experiments and writing the manuscript. MSL performed the pathology analysis. DG prepared the statistical analysis plan and performed the statistical analysis. DWM and EAP participated in data analysis and writing the manuscript. Zhirui W and BMH, as cocorresponding authors, designed and supervised the project, analyzed the data, and wrote the manuscript.

## Supporting information


**Fig. S1.** Liver necropsy gross examination of representative tumor‐bearing mice on Day 22.
**Fig. S2.** Repeat of *in vivo* efficacy study of CCR4‐IL2 IT versus brentuximab using an immunodeficient mouse tumor model.

## Data Availability

The data that support the findings of this study are available from the corresponding author [zhirui.wang@cuanschutz.edu] upon reasonable request.
