# Supplementary material for: CCR4‐IL2 bispecific immunotoxin is more effective than brentuximab for targeted therapy of cutaneous T‐cell lymphoma in a mouse CTCL model
Source: FEBS Open Bio. 2023 May 15;13(7):1309–19. doi: 10.1002/2211-5463.13625 (PMC10315799; doi:10.1002/2211-5463.13625)
Supplement: Supplementary file 1 — Fig. S1. Liver necropsy gross examination of representative tumor‐bearing mice on Day 22. Fig. S2. Repeat of in vivo efficacy study of CCR4‐IL2 IT versus brentuximab using an immunodeficient mouse tumor model. [file FEB4-13-1309-s001.pdf]

Figure S1

CCR4-IL2 IT | CCR4 IT | IL2 IT | Brentuximab full dose | CCR4-IL2 IT + Brentuximab full dose, combination | C21 IT | Brentuximab matching dose

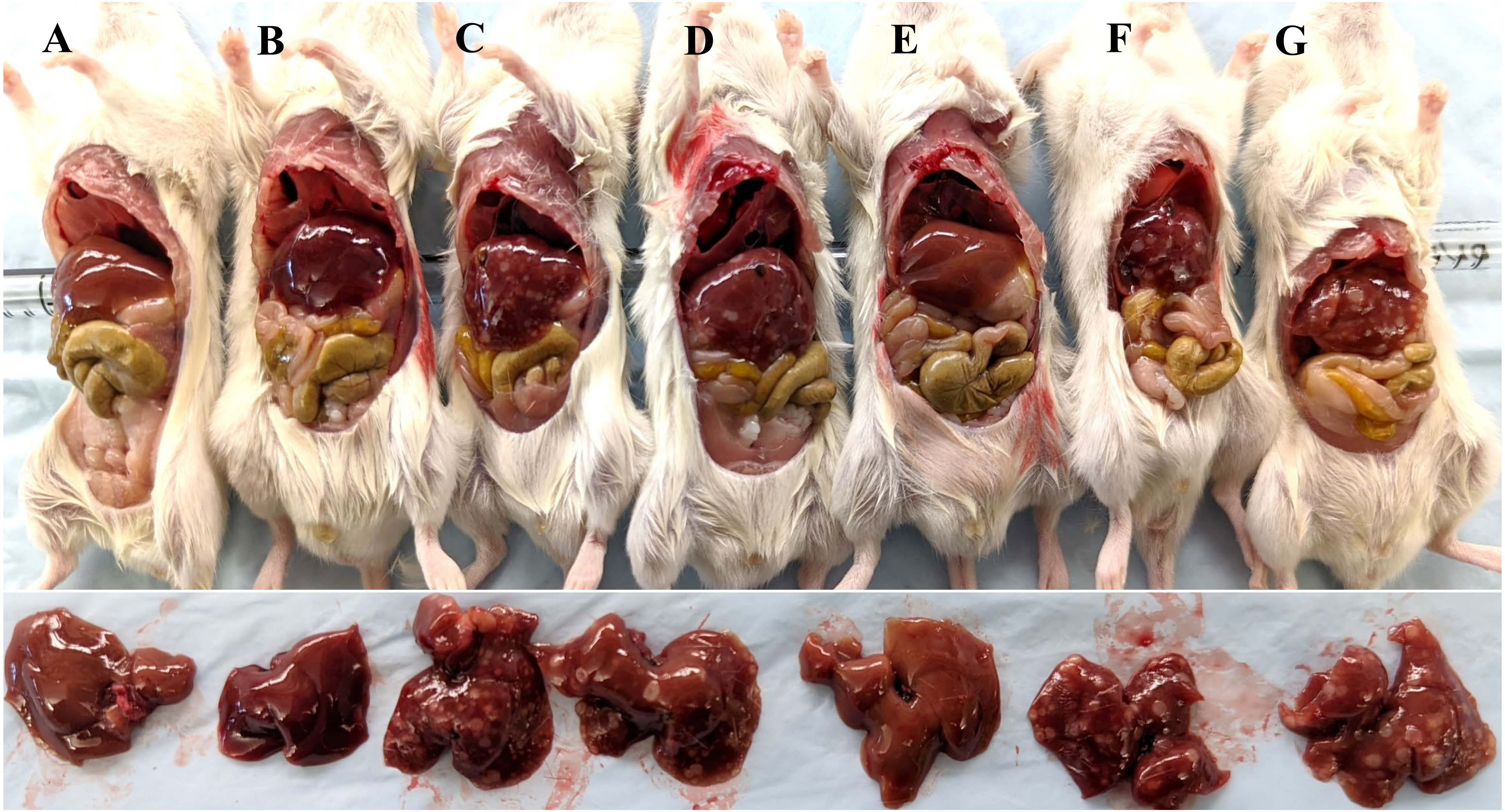

Figure S1. Liver necropsy gross examination of representative tumor-bearing mice on day 22. **A)** CCR4-IL2 IT group. **B)** CCR4 IT group. **C)** IL2 IT group. **D)** Brentuximab full-dose group. **E)** Combination of CR4-IL2 IT and brentuximab full-dose group. **F)** C21 IT group. **G)** Brentuximab matching-dose group. C21 IT, C21 immunotoxin; CCR4 IT, C–C chemokine receptor type 4 immunotoxin; CCR4-IL2 IT, CCR4-interleukin 2 bispecific immunotoxin; IL2 IT, IL2 fusion toxin.

Figure S2

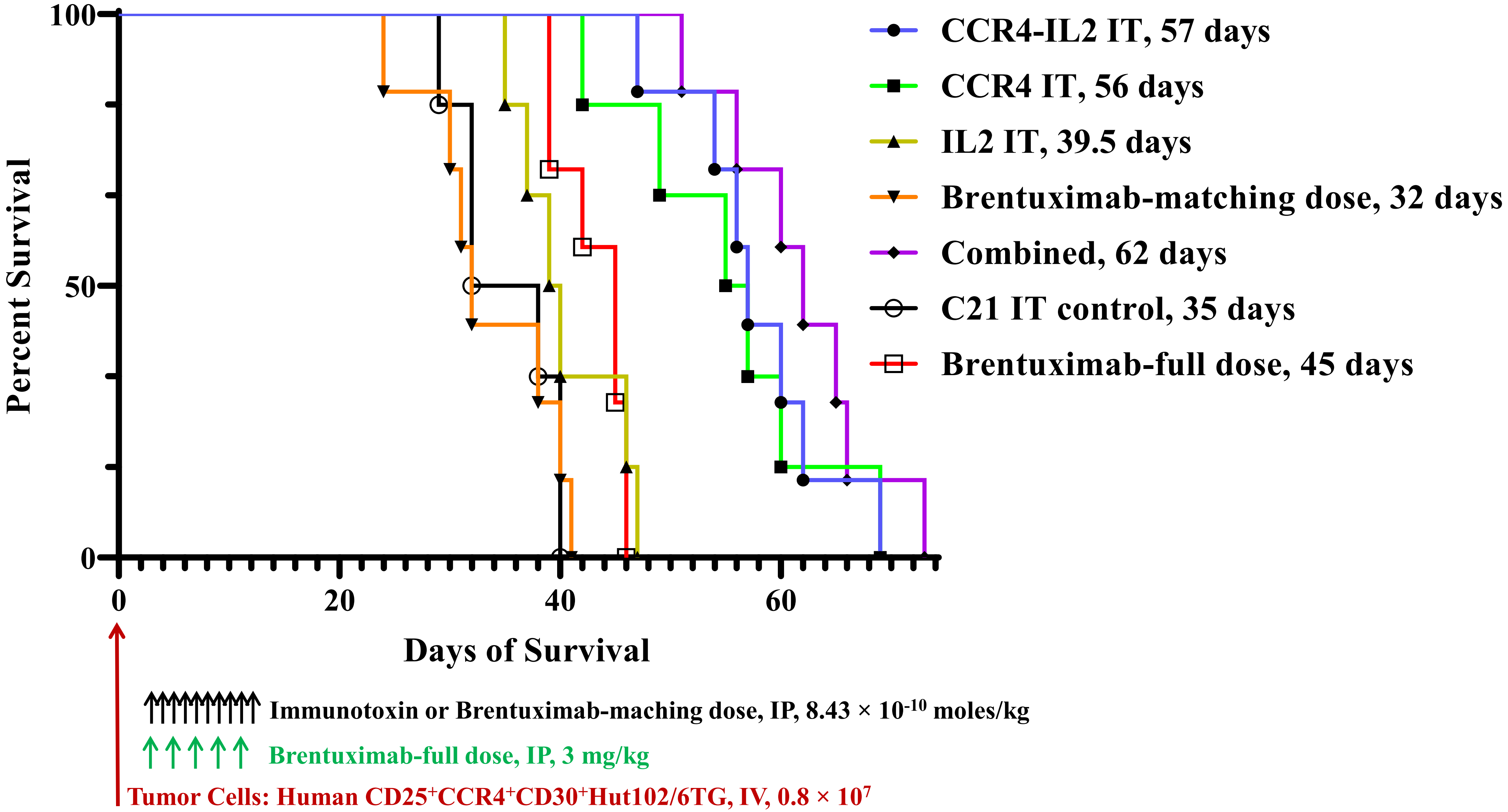

Percent Survival
100
50
0
0
20
40
60
Days of Survival
CCR4-IL2 IT, 57 days
CCR4 IT, 56 days
IL2 IT, 39.5 days
Brentuximab-matching dose, 32 days
Combined, 62 days
C21 IT control, 35 days
Brentuximab-full dose, 45 days
Immunotoxin or Brentuximab-maching dose, IP, 8.43 × 10-10 moles/kg
Brentuximab-full dose, IP, 3 mg/kg
Tumor Cells: Human CD25+CCR4+CD30+Hut102/6TG, IV, 0.8 × 107

Figure S2. Repeat of *in vivo* efficacy study of CCR4-IL2 IT versus brentuximab using an immunodeficient mouse tumor model. Immunodeficient *NSG* mice were IV injected with 8×10^6 $CD25^+CCR4^+CD30^+$ Hut102/6TG cells on day 0. Immunotoxin (CCR4-IL2 IT, CCR4 IT, IL2 IT or C21 IT) or a brentuximab matching dose was IP injected starting on day 4 at 8.43×10^{-10} moles/kg, once daily for 10 consecutive days (10 doses in total). A brentuximab full dose was IP injected starting on day 4 at 3 mg/kg, once every other day for 10 consecutive days (5 doses in total). For combination treatment, CCR4-IL2 IT was IP injected at 8.43×10^{-10} moles/kg, once daily for 10 consecutive days (10 doses in total) and a brentuximab full dose was IP injected at 3 mg/kg, once every other day for 10 days (5 doses in total). CCR4-IL2 IT group (n=7, blue curve) with a median survival time of 57 days. CCR4 IT group (n=7, green curve) with a median survival time of 56 days. IL2 IT group (n=7, light yellow curve) with a median survival time of 39.5 days. Brentuximab matching-dose group (n=7, orange curve) with a median survival time of 32 days. Combination treatment of CR4-IL2 IT and brentuximab full-dose group (n=7, purple curve) with a median survival time of 62 days. C21 IT group (a non-related DT390-based immunotoxin as negative control) (n=7, black curve) with a median survival time of 35 days. Brentuximab full-dose group (n=7, red curve) with a median survival time of 45 days. The schedule of the IP injection of the immunotoxin or brentuximab, and IV injection of tumor cells are pictured in the schematic below the survival curve. The vertical arrows indicate the days on which tumor cells (red arrows), immunotoxin, brentuximab matching dose (black arrows) or brentuximab full dose (green arrows) were injected. C21 IT, C21 immunotoxin; CCR4 IT, C–C chemokine receptor type 4 immunotoxin; CCR4-IL2 IT, CCR4-interleukin 2 bispecific immunotoxin; IP, intraperitoneally; IL2 IT, IL2 fusion toxin; IV, intravenously.
